# Choroidal Neovascular Membranes in Retinal and Choroidal Tumors: Origins, Mechanisms, and Effects

**DOI:** 10.3390/ijms24021064

**Published:** 2023-01-05

**Authors:** Federico Di Staso, Mariachiara Di Pippo, Solmaz Abdolrahimzadeh

**Affiliations:** 1 Ophthalmology Unit, Neurosciences, Mental Health and Sensory Organs (NESMOS) Department, Sapienza University of Rome, 00100 Rome, Italy; 2 Connecticut Uveitis Foundation, 1043 Farmington Avenue, West Hartford, CT 06107, USA; 3 St. Andrea Hospital, Via di Grottarossa 1035/1039, 00189 Rome, Italy

**Keywords:** choroidal neovascularization, tumor, choroid, vascular endothelial growth factor, VEGF, nevus, melanoma, hemangioma, osteoma

## Abstract

Choroidal neovascularizations are historically associated with exudative macular degeneration, nonetheless, they have been observed in nevus, melanoma, osteoma, and hemangioma involving the choroid and retina. This review aimed to elucidate the possible origins of neovascular membranes by examining in vivo and in vitro models compared to real clinical cases. Among the several potential mechanisms examined, particular attention was paid to histologic alterations and molecular cascades. Physical or biochemical resistance to vascular invasion from the choroid offered by Bruch’s membrane, the role of fibroblast growth factor 2 and vascular endothelial growth factor, resident or recruited stem-like/progenitor cells, and other angiogenic promoters were taken into account. Even if the exact mechanisms are still partially obscure, experimental models are progressively enhancing our understanding of neovascularization etiology. Choroidal neovascularization (CNV) over melanoma, osteoma, and other tumors is not rare and is not contraindicative of malignancy as previously believed. In addition, CNV may represent a late complication of either benign or malignant choroidal tumors, stressing the importance of a long follow-up.

## 1. Introduction

Choroidal and retinal tumors are relatively rare, but diagnosis and treatment can be quite complex. Their nature presents additional challenges compared to more common ophthalmic diseases: management of an eye tumor often requires a multidisciplinary approach with special expertise and equipment, and every effort must be made to save the patient’s life, vision, and the eye [1]. Choroidal neovascularization (CNV) has been reported in association with different retinal and choroidal tumors. Histopathologic studies of choroidal naevi showed that tumoral damage of the choriocapillaris may play a role in CNV development, postulating that choriocapillaris obliteration, along with a compromised mechanical barrier, produces angiogenic factors and sets conditions for CNV induction [2,3]. CNV formation has been observed in several tumoral conditions: on longstanding benign lesions, such as nevus [4], over malignant lesions, such as melanoma [5,6], and even years after rare and benign choroidal lesions, such as choroidal osteoma [7]. This literature review aims to analyze histological characteristics, molecular features, complications, and implications of CNV associated with the most common eye tumors. 

## 2. Results

### 2.1. Choroidal Neovascularization Mechanism in In-Vivo and In-Vitro Models

**Figure 1:**
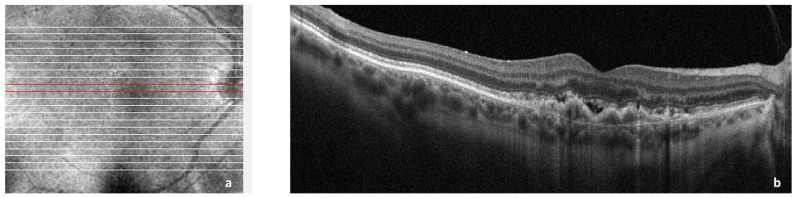
Spectral domain optical coherence tomography (SDOCT) of the macular area showing choroidal neovascularization (CNV) secondary to age-related macular degeneration. (**a**): Image of the macula showing overlying raster scan; (**b**): cross-sectional SDOCT scan showing retinal pigment epithelium (RPE) elevation due to CNV underlying the RPE and adjacent subretinal fluid.

CNV is historically associated with exudative macular degeneration, which is characterized by the deposition of insoluble material in the retinal layers, choriocapillaris thinning, and alteration in Bruch’s membrane thickness. There is an accumulation of lipoproteins, which leads to retinal pigment epithelium (RPE) and photoreceptor atrophy, para-inflammation, and hypoxia. Together, they eventually lead to the secretion of vascular endothelial growth factor (VEGF) from RPE, photoreceptors, and immune cells, which promotes neovascularization [8,9,10]. Exudative age-related macular degeneration (AMD) is a consequence secondary to CNV [11] as shown in Figure 1.

The first classification of the different types and growth patterns was built in 1991 around fluorescein angiography (FA) leakage evidence dividing the neovascular membranes in occult or classic [12]. The occult neovascular membrane is an ingrowth of a neovascular complex initially from the choriocapillaris, into and within the sub-RPE space, that shows a stippled hyperfluorescence, which expands and becomes more evident only in the later phases of FA; classic neovascular membrane originates from the choroid and traverses Bruch’s membrane and the RPE; hence, it may be detected in the very early phase of FA [11,12]. In the following years, the histologic subtypes were classified as CNV type 1, where the CNV is located below the RPE, and CNV type 2, where the CNV is located above the RPE to describe occult and classic neovascularization, respectively [11]. Both are mainly composed of fibrovascular tissue. A combined subtype of type 1 and 2 was also identified [13,14].

Even if the exact mechanisms of AMD remain obscure, the use of experimental models is progressively enhancing our understanding of AMD etiology. Numerous in vivo and in vitro models have been created to reproduce pathogenetic conditions and, despite limitations, existing animal and cellular models are currently uncovering important elements of vascular-related molecular mechanisms in CNV/AMD [15]. Choroidal neovascularization can be reproduced in animal models performing a breach to the integrity of Bruch’s membrane. This can be achieved using laser photocoagulation to generate burns and subsequently observing CNV formation [16,17]. These animal models have been used to explore the various molecular mechanisms of CNV and potential pharmacological implications [18,19]. The importance of VEGF signaling in the development of CNV gradually emerged [20]. In past studies, it was noted that expression of fibroblast growth factor 2 (FGF) and VEGF is increased in association with CNV [21,22], but further studies demonstrated that FGF2 is not necessary for the development of CNV [23]. On the other hand, inhibition of VEGF receptor tyrosine kinase activity drastically inhibits CNV, pointing to VEGF as a main stimulator for CNV in mice [20]. It is interesting to note that transgenic mice expressing higher than normal levels of VEGF in photoreceptors, show neovascularization originating from retinal vessels, but never from choroidal vessels [20]. Normal RPE cells and/or Bruch’s membrane may provide physical or biochemical resistance to vascular invasion from the choroid. This evidence is supported by observing that photoreceptor degeneration results in increased expression of VEGF in RPE cells [24]. These findings suggest that RPE cells may increase VEGF production when exposed to certain extracellular matrix components, and CNV formation may be stimulated by RPE-derived VEGF with physical access to choroidal vessels. Additionally, other studies reported apelin and tumor growth factor-β (TGF-β) signaling to play an essential role to stimulate CNV in mouse models [25] along with growing evidence of Yes-associated protein (YAP) found to promote CNV by stimulating the proliferation of endothelial cells [26]. Despite known limitations of in vivo models, such as anatomical differences between mice and humans (absence of macula in mice), or laser-induced direct damage to the neural retina, which can trigger neuroretinal changes and remove biological overlapping between experimental CNV and human CNV [27], recent advances have been made with nanotechnology in mice models for treating CNV [15]. In vitro cellular systems are widely used as a model for hypothesis testing due to their possibility to study specific cell type effects without confounding factors present in in vivo models [15]. However, human- and animal-derived choroidal endothelial cells are a relatively rare resource that can only be collected post-mortem. Additionally, there is an intrinsic difficulty in maintaining endothelial identity in long-term cell cultures [28]. To overcome those technical difficulties, a recent study discovered a way to immortalize human choroid endothelial cells (CEC) using an endothelial cell-specific promoter, CDH5p-hTERT/CDH5p-Tag. Immortalized CEC lines may offer a more reliable in vitro model, given the expression of endothelial-specific markers such as vWF, CD34, choroid-restricted marker carbonic anhydrase IV along with endothelial functional features [29]. Another step towards a closer anatomical association has been the combined culture of choroidal endothelial cells with RPE cells [30]. 

### 2.2. Vascular Mechanisms in Choroidal Neovascularization

The first and leading hypothesis in 1987 was that all new vessels in CNV arise from pre-existing choroidal vasculature [31]. In the 1990s, bone marrow circulating progenitor cells were identified as contributors to adult vasculogenesis [32,33]. With the laser photocoagulation-induced injury to the choroid described above, researchers transplanted enhanced green fluorescent protein (EGFP)-expressing bone marrow cells from EGFP donor mice into laser-treated mice. Green fluorescent protein recruited cells (GFP+) were quantified in the choroidal vasculatures or in Bruch’s membrane injury sites, showing different levels of contribution in CNV [34,35]. The proportion of GFP+ cells contributing to lesion endothelial cells was observed to be dependent on the stage of CNV [36,37], and mobilized adult hematopoietic stem cells were observed to be able to form endothelial cells subsequently incorporated into choroidal neovasculature [38]. Similar hemangioblast activity was also observed in the murine model [39] and in humans. In the latter, the presence of bone marrow-derived progenitor was identified in excised CNV sections by tracing the AC133 marker of hematopoietic stem cells and bone marrow-derived progenitors [40].

According to some authors, a vascular injury is one of the first steps required for the mobilization of hematopoietic cells. Choroidal vascular damage may release molecular signals that guide the recruitment of circulating progenitor cells, once in situ, differentiation into vascular endothelial and smooth muscle cells occur [37]. The entire process seems to have four phases: mobilization, migration, adhesion, and differentiation [41]. As a consequence of local tissue injury, various cytokines levels such as VEGF, granulocyte colony-stimulating factor (G-CSF), and erythropoietin (EPO) increase. Raising cytokine levels stimulates metallopeptidase 9 (MMP9) activation and triggers the release of bone marrow cells from the bone marrow. Among chemokine mediators, the chemoattractant stromal-derived factor (SDF-1) is expressed by RPE after a laser injury and binds the CXCR4 receptor on bone marrow cells. The chemotactic gradients then guide the migration of bone marrow cells to the local neovascular lesion site [42]. Vascular cell adhesion molecule-1 (VCAM-1) and intercellular adhesion molecule-1 (ICAM-1) then allow the adhesion of migrated bone marrow cells to pre-existing endothelial cells at the site of interest. The last phase is the local differentiation of the bone marrow progenitors into endothelial cells, smooth muscle cells, and macrophages. On the other hand, several studies refute the bone marrow cells contribution hypothesis. Okuno et al. in 2011 [43], showed that bone marrow-derived cells did not take part in the healing process as differentiated endothelial cells, but mainly as pro-angiogenic macrophages. Accordingly, it was demonstrated that during vasculogenesis, none of the bone marrow-derived cells contributed to the endothelium and in vivo endothelial differentiation rarely occurred. Specifically, no bone marrow-derived VEGFR-2+ or other endothelial cell precursors were observed to contribute to vascular endothelium; hence, even cancer growth does not require bone marrow-derived endothelial progenitors [44]. In another study, resident stem-like/progenitor cells were identified in pre-existing endothelium, showing proliferation ability [45]. Wakabayashi et al. [46] found that these progenitors, defined as “endothelial side population cells” did not originate from the bone marrow. Furthermore, when isolated from murine choroidal tissue, the endothelial side population showed exceptional colony-forming ability in vitro along with increased proliferation in laser-induced choroidal neovascularization in vivo. These results taken together, highlight that endothelial cells participating in CNV could potentially originate from circulating bone marrow progenitors, circulating hematopoietic stem cells, and vessel-residing endothelial side population cells. 

### 2.3. Molecular Mechanisms

**Figure 2:**
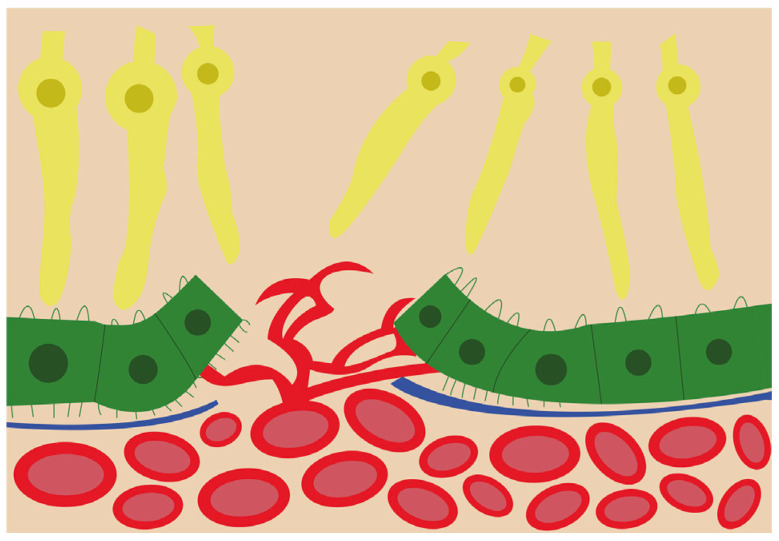
Schematic view of the choroidal neovascularization process. The new blood vessels from the choroid have breached the retinal pigment epithelium and are branching and invading the retina.

VEGF is found in several isoforms, VEGF_121_, VEGF_145_, VEGF_165_, VEGF_189_, and VEGF_206_, and is a strong angiogenic molecule capable of stimulating proliferation, migration, and enhancing vascular permeability of endothelial cells [47,48]. Dysregulation of VEGF is known as one of the main steps in pathological angiogenesis [49]. In physiological conditions, VEGF and FGF2 are released by the RPE during fetal development to promote the development of the choriocapillaris [50]. Furthermore, VEGF is required for the formation of fenestrations in the choriocapillaris [51] in order to allow macromolecule transit in and out of choroidal circulation [50]. Under normal conditions, VEGF is kept at a basal level, but in pathological reactive conditions, such as CNV, VEGF levels are significantly increased [49]. The VEGF isoform that is predominantly involved in pathological angiogenesis is VEGF_164/165_ [52]_._ In addition to VEGFs’ stimulating role in angiogenesis, their elevated level in the RPE leads to barrier disruption, which could further increase neovascularization chances [51] as illustrated in Figure 2.

Many other molecular mechanisms are currently identified as possible co-factors in AMD CNV; nevertheless, such mechanisms have never been related to tumoral settings. By way of example, high-temperature requirement A serine peptidase 1 (HTRA1), is a multi-functional serine protease that regulates vascular growth and is essential for normal vasculature development in the brain and eye [53]. It was suggested that high levels of HTRA1 may compromise the integrity of Bruch’s membrane, creating a way for choroidal vasculature infiltration [54]. Even antioxidants have been found to slow the progression of AMD, calling attention to the role of oxidative stress. An altered Bruch’s membrane may trigger the development of CNV while oxidative stress may set pro-angiogenic conditions [55]. In addition, oxidative stress is also known to concur in precocious aging of the RPE [56] and the latter has been found to increase the expression of VEGF, contributing to CNV [57,58]. Despite these studies having shown the role of oxidative stress in choroidal angiogenesis, there is not enough evidence of oxidative stress’s role in a tumoral-associated CNV. Anti-VEGF is the mainstay of CNV management in AMD, often requiring lifelong treatment [59,60,61]. Typically, the initial visual improvement of the first few months is followed by a plateau phase that may last throughout the entire course of treatment, this effect is known as anti-VEGF resistance. In tumor studies, anti-VEGF resistance has been associated with the secretion of platelet-derived growth factor (PDGF) by tumor cells [62]. PDGF stimulates the recruitment and proliferation of pericytes to neovascular segments and this subsequently stabilizes endothelial cells. Furthermore, pericytes induce the antiapoptotic protein Bcl-w in tumoral endothelium, both in vivo and in vitro, thereby granting protection from cytotoxic damage; this is believed to be the start of an autocrine loop involving VEGF-A expression in endothelial cells and a logical explanation for anti-VEGF resistance [63]. In a choroidal neovascular site, edge cells forming the vascular advancing front express PDGF, which in turn causes the recruitment of pericytes and microvessel maturation. Those recruited pericytes form a barrier around the new endothelium reducing the effect of VEGF inhibitors and explaining the plateau phase in anti-VEGF treatment [63,64].

### 2.4. CNV Associated with Choroidal Nevi and Melanomas

Choroidal nevi are benign pigmented tumors that can only occasionally cause loss of visual acuity [65]. Over time, these lesions may induce secondary changes in the pigment epithelium and lead to the formation of drusen, serous retinal detachment, and proliferation of CNV [66]. In 2004, Zografos et al. [67] described 22 cases of choroidal nevi inducing the formation of a neovascular membrane. The CNV was situated close to the center of the pigmented nevus, was not larger than the nevus in 20 cases, and extended beyond the edge of the lesion in the other 2 cases. CNV was classic in all cases. 

Choroidal melanomas are the most common primary intraocular malignancy in adults [68]. The Collaborative Ocular Melanoma Study (COMS) found that choroidal melanomas are more common in Caucasians and that the mean age at diagnosis is 60 years [69]. These melanomas are usually located posterior to the ciliary body. Typically asymptomatic, they are most commonly found during routine ophthalmic examination. When symptomatic, depending on the tumor location, they can induce “flashing lights” due to tumor-induced exudative retinal detachment or metamorphopsia due to subfoveal tumor [70]. 

Accumulations of lipofuscin and melanolipofuscin as yellow/orange pigment can be visualized on the surface of the melanoma. An exudative subretinal fluid can also be also observed overlying the primary tumor, indicating incontinent tumor blood vessels leaking beneath the retina [70]. Lubin et al. in 1982 [5], described a case of malignant choroidal melanoma associated with CNV, becoming the first histologic verification to appear in the literature. Guerin et al. in 2006 [71], examined a series of choroidal melanoma to study the frequency and particular histological tumor characteristics in melanoma-associated CNV. Microscopic evidence of CNVs was found in 14 of the 229 globes examined. The overall incidence was therefore 6%. Each case was examined after multiple sections from different planes were taken and stained with hematoxylin and eosin (H&E), diastase-periodic acid Schiff (DPAS), and Gomori Trichrome (GOM). Three CNVs were located over the tumor apex, two at both sides of the apex, six at the side of the tumor, and the last three over the tumor edge. In 2013, an unusual case of CNV complicating a choroidal nevus in a 16-year-old patient was also reported [72]. 

### 2.5. CNV in Choroidal Osteoma

Choroidal osteoma is a benign intraocular tumor made of mature bone that replaces the entire choroid. This tumor typically appears as a yellow–orange plaque on retinal examination. Usually found in the juxta papillary or macular region [73], it manifests as a unilateral lesion in young females. The etiology and pathogenesis of choroidal osteoma are poorly understood, but after many years and several studies, it was eventually recognized to be related to CNV [74,75]. In 1998, Aylward et al. [76] observed the long-term outcome of 36 patients with choroidal osteoma, showing a moderate risk for the development of CNV. Several years later, in 2005, Shields et al. [77] conducted an extensive retrospective nonrandomized study evaluating choroidal osteoma for tumor growth, tumor decalcification, and CNV. CNV was associated with a choroidal osteoma in 21% of eyes on 1-year follow-up, but in 46% of eyes on 20-year follow-up. The greatest risk for the development of CNV was an irregular surface and an overlying hemorrhage. Other case reports corroborated the correlation between choroidal osteoma and the development of CNV after several years [7] or even in the case of bilateral osteomas [78]. CNV complicating a choroidal osteoma is typically “classic”, as previously described, but Kim et al. in 2020 [79], described a case of polypoidal choroidal vasculopathy (PCV), which is characterized by more complex and aggressive branching choroidal vessels with terminal (polyp-like) aneurysmal dilations. The authors observed irregular RPE elevations over the regions of decalcification around the optic nerve, suggesting that a quiescent CNV can progress to PCV.

### 2.6. CNV in Choroidal Hemangioma

CNV is a rare event in association with circumscribed choroidal hemangioma (CCH) or after its treatment. Ruby et al. in 1992 [80], reported two patients with choroidal hemangiomas developing CNV; one patient had Sturge–Weber syndrome (SWS) with a unilateral diffuse choroidal hemangioma (DCH). Shields et al. in 2001 [81], observed that only three CCH patients had concomitant CNV in 200 cases examined. An even rarer event was observed in a CCH with HIV infection by Hua et al. in 2014 [82]. Given the case, overall complexity, and the wide range of cofactors involved the authors suggested that various mechanisms may be responsible for the CNV formation. In particular, the authors considered that a continuous vascular leakage of CCH may facilitate angiogenesis led by plasma proteins and fibrin. Furthermore, CCH could stimulate the release of angiogenic factors, due to the constant low-grade inflammation and ischemia. Another possibility is that CNV can also be stimulated by laser-induced necrosis of the tumor and VEGF released after photo-dynamic treatment (PDT). Several complications including RPE alterations and photoreceptor loss have been described after PDT therapy. Despite some evidence showing the development of retinal neovascularization [83] and PCV [84] after PDT, its role in CCH-related CNV is still unclear. 

A peculiar case is represented by the SWS, a neuro-oculo-cutaneous hemangiomatosis where DCH is a key finding [85]. In SWS, the eye is involved in more than 50% of patients and the main features are glaucoma and DCH with several possible complications [86]. SWS genetic studies showed that DCH occurs sporadically from an activating mutation in *GNAQ* at codon R183 [87]. Mutations in *GNAQ* or *GNA11* result in the upregulation of the mitogen-activated protein kinase, which in turn results in cellular proliferation [88]. In 2019, Bichsel et al. [89] showed that the mutation found in most sporadic capillary malformations, *GNAQ* R183Q, was present in the choroidal vessels at a similar frequency to that found in SWS brain tissue, suggesting an analogous choroidal capillary malformation cause. Anti-VEGF use alone is neither resolutive nor indicated, but recent publications are showing the utility of adding anti-VEGF agents to PDT to counter the effect of PDT-induced high VEGF levels [90]. These results indicate that CNV and choroidal capillary malformations follow different pathways, but the development of new vessel complexes in SWS could still represent a potential complication [80]. 

### 2.7. CNV in Primary Vitreous Retinal Lymphoma

Primary vitreous retinal lymphoma (PVRL) is a rare intraocular malignancy and CNV has not been reported regularly as a complication. Ma et al. in 2020 [91], described a case of PVRL characterized by subretinal hyperreflective material and complicated with CNV. Of note, the authors admitted that the CNV could have existed since the first visit but was not recognized on instrumental imaging due to RPE perturbations. Furthermore, these authors hypothesized that the growth of CNV could be related to RPE hypoxia. Lymphoma cells infiltrate and proliferate under the retina producing elevated levels of IL-10 [92]. Il-10 serves as a growth factor of B-cells and the resulting massive cell aggregates under the RPE may affect oxygen diffusion. The natural response would be an upregulation of VEGF. Unfortunately, the evidence in the literature is so scarce that it is impossible to determine a definitive correlation between PVRL and CNV at the moment. 

## 3. Discussion

Proliferation of a CNV in the setting of choroidal tumors is a rare and poorly understood event. Many factors appear to be involved and CNV is considered to be a response to breaches in Bruch’s membrane or to degenerative damages to the RPE–chorioretinal complex. Another factor may be the subtle retinal ischemia secondary to disruption of the choriocapillaris. According to the leading hypothesis in AMD studies, a healthy RPE cell layer prevents VEGF from getting to choroidal vessels from the retina. Accordingly, normal RPE cells and/or Bruch’s membrane may provide physical or biochemical resistance to vascular invasion from the choroid. Oxidative stress may concur in precocious aging of the RPE and increase the expression of VEGF. Furthermore, PDGF stimulates the recruitment and proliferation of pericytes to neo-vascular segments. Stabilized endothelial cells also benefit from the antiapoptotic protein Bcl-w released by recruited pericytes. The factors and mechanisms inducing CNV in melanoma, naevi, and other choroidal tumors may be similar to those involved in AMD, as they can cause morphologically interchangeable RPE changes [93,94]. In addition to the expected angiogenic drive that naturally arises from tumor-induced RPE damage, we should consider angioregulatory factors secreted by the tumor itself. For example, uveal melanoma express pro-angiogenic factors such as VEGF and FGF [95]. Even matrix metalloproteinases are implicated in angiogenesis and are actively secreted by uveal melanoma [96]. Despite this, Guerin et al. [71] showed that the angiogenic promoters in a choroidal melanoma setting can produce larger CNV than in other etiologies, but those are not sufficient conditions to increase the frequency of CNV over that found in choroidal naevi.

CNV over melanoma, osteoma, and other tumors is not rare and is not contraindicative of malignancy as previously believed [4]. In addition, CNV may represent a late complication of either benign or malignant choroidal tumors, stressing the importance of a long follow-up. Further studies are warranted to establish a decisive connection between choroidal tumors and CNV formation, as they could shed new light on tumoral and non-tumoral angiogenic mechanisms. 

## Data Availability

Not applicable.
